# Fabrication of a free-standing Ti_3_C_2_T_*x*_-PTh counter electrode *via* interfacial polymerization for dye-sensitized solar cells†

**DOI:** 10.1039/d4ra02651a

**Published:** 2024-07-31

**Authors:** Suruthi Priya Nagalingam, Saravanan Pandiaraj, Khalid E. Alzahrani, Abdullah. N. Alodhayb, Andrews Nirmala Grace

**Affiliations:** a Centre for Nanotechnology Research, Vellore Institute of Technology Vellore 632014 India anirmalagladys@gmail.com; b Department of Self-Development Skills, King Saud University Riyadh 11451 Saudi Arabia; c Department of Physics and Astronomy, College of Science, King Saud University Riyadh 11451 Saudi Arabia

## Abstract

The current work involves the fabrication of a MXene-Polythiophene (Ti_3_C_2_T_*x*_-PTh) composite *via* interfacial polymerization, alongside its deployment as a counter electrode (CE) or photocathode in dye-sensitized solar cells (DSSCs). The structural properties of the synthesized materials were investigated through a comprehensive array of techniques, including X-ray diffraction (XRD), fourier-transform infrared (FT-IR) spectroscopy, high resolution scanning electron microscopy (HRSEM), energy-dispersive X-ray analysis (EDAX), and X-ray photoelectron spectroscopy (XPS). The electrochemical performance, assessed *via* cyclic voltammetry (CV) and electrochemical impedance spectroscopy (EIS), revealed that the Ti_3_C_2_T_*x*_-PTh CE exhibits superior electro-catalytic activity, and reduction in charge transfer resistance compared to other individual CEs. These observations are in concordance with the data obtained from Tafel analysis. The incorporation of Ti_3_C_2_T_*x*_ sheets into the composite significantly augmented its catalytic efficacy for triiodide reduction, manifesting in elevated short-circuit photocurrent density and enhanced fill factor metrics. A DSSC utilizing the Ti_3_C_2_T_*x*_-PTh CE exhibited a power conversion efficiency (PCE) of 5.83%, which stands on par with that of traditional Pt CEs. Thus, the Ti_3_C_2_T_*x*_-PTh CE material is posited as a viable, cost-efficient alternative to Pt, heralding a new era in the engineering of counter electrodes for the next generation of DSSCs.

## Abstract 2

The current work involves the fabrication of a MXene-Polythiophene (Ti_3_C_2_T_*x*_-PTh) composite *via* interfacial polymerization, alongside its deployment as a counter electrode (CE) or photocathode in dye-sensitized solar cells (DSSCs).

## Graphical abstract

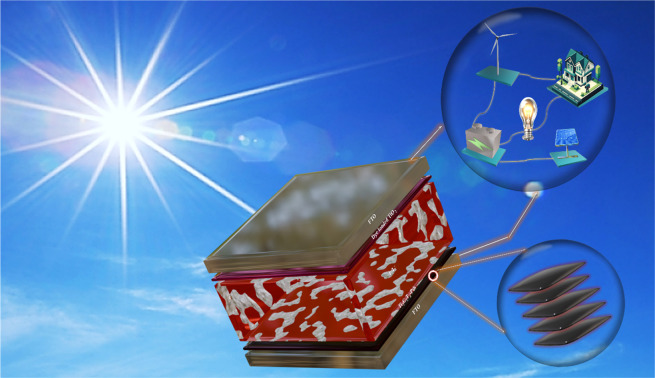

## 1. Introduction

The pressing need to address the impacts of climate change and meet the growing global energy demand requires a revolutionary shift in energy production systems. Solar energy, renowned for its renewable, pristine, and limitless characteristics, emerges as a possible remedy in the quest for a low-carbon economy.^1–3^ Since their invention in 1991 by Gratzel's group, dye-sensitized solar cells (DSSCs) have become a viable third-generation choice among several solar cell technologies, known for their cheap cost, high efficiency, and simple production procedure.^4,5^ DSSCs generally consist of the following components: a photocathode, a dye-sensitized titanium dioxide (TiO_2_) photoanode, coupled with an electrolyte comprising a redox pair of iodide and triiodide redox pair.^6^ The counter electrode (CE) is of utmost importance as it facilitates the catalytic reduction of triiodide ions by collecting electrons from the external circuit. Due to its outstanding catalysis and electron exchange properties, platinum (Pt) has long been considered the gold standard for CE materials; yet, the high cost and limited availability of Pt are formidable obstacles to the commercialization and mass adoption of DSSCs.^7,8^ As a result of this constraint, investigations have been initiated into non-Pt CE materials that are environmentally friendly and cost-effective, and which can match or surpass the performance of Pt.^9,10^ To replace Pt, a number of contenders have showed promise, including composites, metal alloys,^11^ carbon-based materials,^12,13^ transition metal compounds,^14^ and conductive polymers.^15,16^ These developments are in line with the worldwide movement towards renewable energy sources and represent a larger effort to create DSSCs with improved environmental sustainability, lower prices, and greater power conversion efficiencies (PCE).

MXenes (Ti_3_C_2_T_*x*_), characterized by their two-dimensional (2D) structures of transition metal nitrides, carbonitrides, and carbides have emerged as prominent materials in energy storage and conversion technologies.^17,18^ These materials boast significant surface area, high electrical conductivity, a wide range of compositions, and the ability to undergo surface modifications. The layered structure of Ti_3_C_2_T_*x*_, coupled with their excellent ion intercalation capability, thermal stability, flexibility, and modifiable surface terminal groups (such as hydroxyl, oxygen, and fluorine), renders them a focal point of research across various domains, including wearable electronics, supercapacitors, photovoltaics, and more.^17,19–24^ In the domain of third-generation photovoltaic technologies, Ti_3_C_2_T_*x*_ have shown considerable promise in enhancing the efficiency of DSSCs, perovskite solar cells (PSCs), and quantum dot solar cells (QDSCs).^25^ This enhancement is primarily attributed to Ti_3_C_2_T_*x*_ ability to stabilize the perovskite layer, improve charge carrier mobility, and adjust work functions at different interfaces, including photoanodes, hole transporting layers, and CEs. Notably, Ti_3_C_2_T_*x*_ have been utilized as additives in mesoporous TiO_2_-based photoanodes for DSSCs, significantly improving charge transfer and light scattering capabilities, thereby augmenting light-harvesting and electron collection efficiencies.^26,27^ Furthermore, the conductive nature of Ti_3_C_2_T_*x*_ offers an economically viable and stable alternative to Pt CEs, overcoming the latter's cost and instability challenges in acidic environments. Recent advancements have showcased the potential of Ti_3_C_2_T_*x*_ to surpass conventional Pt-based CEs in terms of PCE. Innovations such as hydrothermal deposition of CoS on Ti_3_C_2_T_*x*_ and the creation of CoS/Ti_3_C_2_T_*x*_ CE through ion-exchange techniques have led to significant improvements in electrocatalytic properties and PCE of 8.0%.^28^ Additionally, incorporating carbon nanotubes (CNTs) to prevent agglomeration has yielded structures with optimal ratios of CoMoP_2_ and Ti_3_C_2_T_*x*_, achieving remarkable PCE enhancements of 10.64%.^29^ These developments highlight Ti_3_C_2_T_*x*_ as cost-effective, high-performing, and stable Pt-free alternatives for CEs in DSSCs. However, challenges such as charge trapping within MXene's multilayered structure and van der Waals force-induced stacking, which hinder electron transport and reduce specific surface area and conductivity, remain.^8,30^ To address these issues, surface modification with conductive polymers like polyaniline (PANI), polypyrrole (PPy), and particularly polythiophene (PTh) and its derivatives has been proposed.^31^ These modifications aim to improve electrochemical performance and stability by preventing stacking and enhancing the electroactive surface area. A novel approach involving the integration of PTh nanoparticles between Ti_3_C_2_T_*x*_ nanosheets has been explored, resulting in the creation of Ti_3_C_2_T_*x*_-PTh hybrid. This method, which involves liquid–liquid interfacial polymerization, offers a simple and room-temperature process for producing high-molecular-weight polymers.^32^ The resulting Ti_3_C_2_T_*x*_-PTh hybrid manifests considerable potential as a CE material for the first time, amalgamating the advantageous properties of its constituent materials. The physicochemical characteristics of the composite, alongside its photovoltaic performance metrics, were systematically delineated. DSSCs fabricated utilizing the Ti_3_C_2_T_*x*_-PTh as the CE, have demonstrated an PCE to 5.82%, equivalently efficacious to that of conventional Pt CE.

## 2. Experimental methods

### 2.1 Synthesis of Ti_3_C_2_T_*x*_ nanosheets

In a meticulously controlled experimental procedure, approximately 0.5 grams of the MAX phase material, Ti_3_AlC_2_, was subjected to an etching regimen utilizing a 30 ml of 40% hydrofluoric acid solution over a span of two days. This etching treatment was succeeded by multiple centrifugation cycles, interspersed with thorough washes using deionized water, to facilitate the isolation of ml-Ti_3_C_2_T_*x*_. Subsequently, the resultant mixture underwent vacuum filtration, followed by a drying phase at 60 °C for an extended period.

### 2.2 Synthesis of Ti_3_C_2_T_*x*_-PTh CE

**Fig. 1:**
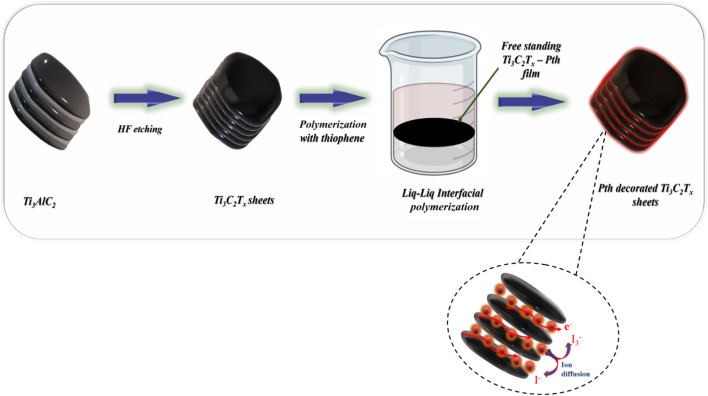
A schematic representation of the synthesis of Ti_3_C_2_T_*x*_-Pth.

As shown in Fig. 1 the fabrication of the Ti_3_C_2_T_*x*_-PTh hybrid was developed *via* a liquid–liquid interfacial polymerization method. The process of liquid–liquid interface polymerization was initiated by first preparing an aqueous solution containing a predetermined quantity of 0.3 g of ferric chloride hexahydrate, and 0.05 g of Ti_3_C_2_T_*x*_, ultrasonically disseminated within 15 ml of distilled water. Following this, the monomeric solution was prepared by thoroughly mixing 0.5 ml of thiophene into 15 ml of chloroform to form the organic solvent phase, an essential step in the process. The aqueous solution was then meticulously added to the organic solution in a dropwise manner, ensuring minimal disturbance to the resultant mixture, which was then left to stand undisturbed for a duration of 24 hours. During this quiescent period, a notable phenomenon was observed wherein a black film gradually emerged at the interface between the aqueous and organic phases, indicating the successful formation of a polymerized film. This observation underscores the critical nature of the interface in facilitating the polymerization process. The culmination of this synthesis process involved the purification of the resultant polymeric material through sequential ethanol washes and subsequent drying at 60 °C for 24 hours. For comparative analysis, PTh was synthesized employing the same methodology, albeit in the absence of the Ti_3_C_2_T_*x*_.

### 2.3 DSSC construction and assembly using various CEs

The cleaning regimen for fluorine-doped tin oxide (FTO) glass substrates incorporated an initial sonication in a detergent solution, followed by successive washings with deionized water, acetone, and isopropanol, ensuring a pristine surface for subsequent processing. The fabrication of the TiO_2_ photoanode commenced with the preparation of a viscous paste, constituted of 230 mg TiO_2_ (P25) nanoparticles, 230 μl deionized water (DI), 10 μl of conc. nitric acid (HNO_3_), and 10 μl Triton-X. This paste was meticulously applied onto a pre-cleaned FTO surface through the application of the doctor blade method, ensuring a uniform layer over a defined area of 4 mm by 4 mm. Subsequent thermal treatment involved sintering the coated substrate at 450 °C for a period of 30 minutes to foster enhanced adhesion properties, followed by a natural cooling process to reach ambient temperature conditions. In the subsequent stage, a dye solution was formulated by dissolving 0.3 mM of the N719 dye in ethanol. The previously prepared TiO_2_ layer was immersed in this solution for an extended duration, specifically overnight, to ensure comprehensive adsorption of the dye molecules. To remove any non-adsorbed dye, the electrode was rinsed with ethanol. CEs, including variants such as Ti_3_C_2_T_*x*_, PTh, and a Ti_3_C_2_T_*x*_-PTh hybrid, were fabricated by creating a thick slurry of the respective CE materials dispersed in an *N*-methyl-2-pyrrolidone medium. This paste was then evenly applied to a precleaned FTO surface using the doctor blade method, targeting an area specification of 4 mm by 4 mm. A sintering process at 80 °C for one hour was then employed, allowing the substrates to gradually return to room temperature. A Pt CE was also prepared by dispersing 10 mM hexachloroplatinic acid in isopropyl alcohol and applying this dispersion to a similar-sized area on the FTO, which was subsequently sintered at 450 °C for a period of 30 minutes before being allowed to cool to ambient temperature. The electrolyte solution for the DSSC was prepared by combining 0.05 M iodine (I_2_), 0.5 M lithium iodide (LiI), and 0.1 M 4-*tert*-butylpyridine in acetonitrile. Assembly of DSSCs entailed positioning the dye-loaded TiO_2_ as the photoanode and the various prepared CEs, with the entire setup secured together. The final step in the assembly process involved the careful injection of the iodide/triiodide electrolyte between the electrodes, completing the DSSC construction.

## 3. Results and discussion

**Fig. 2:**
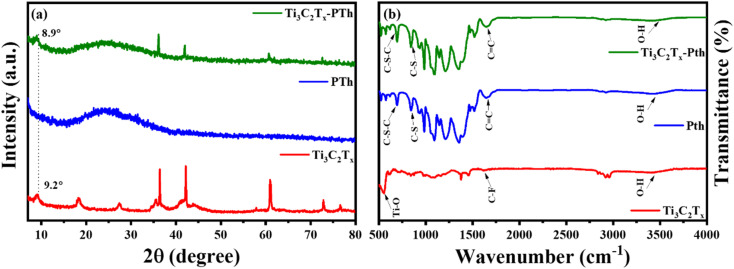
(a) XRD patterns and (b) FT-IR spectra of Ti_3_C_2_T_*x*_, Pth, and Ti_3_C_2_T_*x*_-Pth.

The elucidation of structural modifications in Ti_3_C_2_T_*x*_ through powder XRD analysis highlights significant findings, particularly concerning the crystalline structure alterations post HF etching as shown in Fig. 2(a). Notably, the observed reduction in the peak intensity at 38.9°, which is associated with the (104) plane of Ti_3_AlC_2_, signifies the transition to Ti_3_C_2_T_*x*_. This transformation is evidenced by the pronounced decrease in peak intensity, suggesting the removal of the Al layer and thus, confirming the conversion from the MAX phase to MXene, as supported by existing literature.^33,34^ Additionally, the alteration in the positions of the (002) peak from 9.5° to 9.2° is indicative of an increase in interlayer spacing, further validating the successful etching process.^35,36^ For the analysis of pristine PTh, its XRD pattern presents a broad peak around 22°, reflecting its amorphous nature. When considering the Ti_3_C_2_T_*x*_-PTh hybrid, the XRD analysis reveals both the broad peak corresponding to PTh and a distinct peak indicative of Ti_3_C_2_T_*x*_. The shift of the Ti_3_C_2_T_*x*_ peak from 9.2° to 8.9° in the composite suggests an effective exfoliation of Ti_3_C_2_T_*x*_ within the PTh matrix, leading to an increased *d*-spacing.^33,37^ This observation underscores the successful integration of Ti_3_C_2_T_*x*_ into the PTh matrix through interfacial polymerization, marking a notable structural adaptation and highlighting the intricate interfacial interactions within the composite material. The identification of functional groups within the CE materials was examined *via* Fourier-transform infrared spectroscopy (FT-IR), further enriched by a meticulous analysis of their compositional and structural aspects through the discernment of specific functional group peaks. FT-IR spectral analysis delineated in Fig. 2(b) for Ti_3_C_2_T_*x*_, PTh, and the Ti_3_C_2_T_*x*_-PTh composite revealed distinctive spectral phenomena. For PTh, the spectral analysis identified peaks at 692 cm^−1^, and 830 cm^−1^, which are attributable to C–S–C bending vibrations, and C–S stretching, respectively.^38–40^ These observed peaks corroborate the existence of thiophene rings, thereby confirming the efficacious synthesis of PTh.^40^ In the Ti_3_C_2_T_*x*_ spectrum, an eminent peak at 3440 cm^−1^ was indicative of –OH stretching vibrations, denoting a significant presence of hydroxyl (–OH) groups on the Ti_3_C_2_T_*x*_ nanosheets. Peaks observed at 570 cm^−1^ and 1621 cm^−1^ were correlated with Ti–O and C–F bonds, respectively, accentuating the presence of these functional groups within the Ti_3_C_2_T_*x*_ framework.^40^ In the spectrum for the Ti_3_C_2_T_*x*_-PTh hybrid, absorption peaks characteristic of both constituent materials was identified, demonstrating an extensive integration of PTh into the Ti_3_C_2_T_*x*_ matrix. Notably, the composite material's spectrum exhibited subtle shifts in peak positions and a diminution in peak intensities in comparison to the pristine PTh spectrum.^32^ These modifications suggest an effective intercalation of Ti_3_C_2_T_*x*_ into the polymeric matrix, indicative of alterations in the composite's structural and compositional characteristics and elucidating the intricate interfacial interactions between MXene and PTh within the composite.

**Fig. 3:**
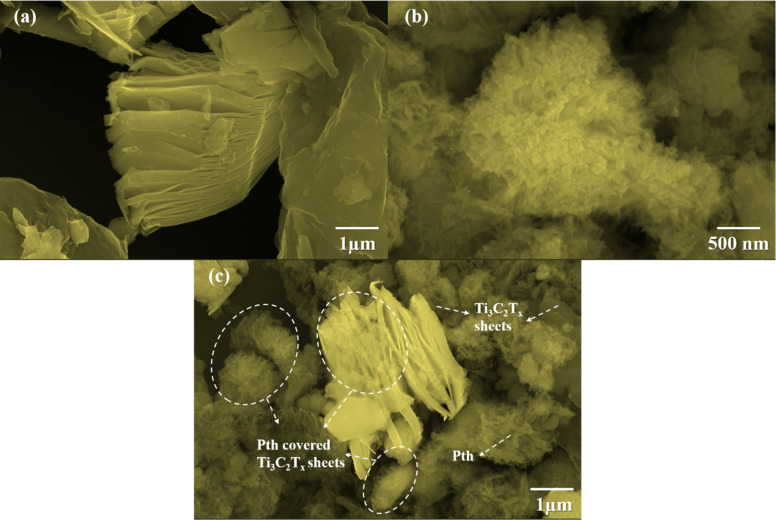
SEM images of (a) pristine Ti_3_C_2_T_*x*_, (b) pristine Pth, and (c) Ti_3_C_2_T_*x*_-Pth.

The investigation of the surface morphologies of Ti_3_C_2_T_*x*_, PTh, and their hybrid Ti_3_C_2_T_*x*_-PTh was undertaken through High Resolution Scanning Electron Microscopy (HRSEM) as given in Fig. 3(a)–(c). This analysis confirmed that Ti_3_C_2_T_*x*_ displays a stratified sheet-like morphology. In contrast, SEM observations of PTh revealed a distinct granular form.^38^ This specific morphology was also detected atop the Ti_3_C_2_T_*x*_ layers within the composites, indicating a successful adherence and polymerization of PTh onto these sheets. To complement these findings, Energy Dispersive Spectroscopy (EDS) elemental mappings were employed, showcasing a uniform distribution of the S element across the Ti_3_C_2_T_*x*_ structures as shown in Fig. S1 and S2.† This distribution mirrors the SEM results, underscoring an even spread of PTh throughout the composite material. In essence, the combined SEM and EDS investigations elucidate a porous architecture within the Ti_3_C_2_T_*x*_-PTh composite, facilitating effective ion and electron transport. This structure is attributed to the various functional groups serving as nucleation points during PTh's polymerization on the Ti_3_C_2_T_*x*_ sheets. Therefore, the synthesized composite material is characterized by an expansive surface area and multiple pathways for electrolyte ions, positioning it as a promising candidate for utilization in diverse electrochemical systems.

**Fig. 4:**
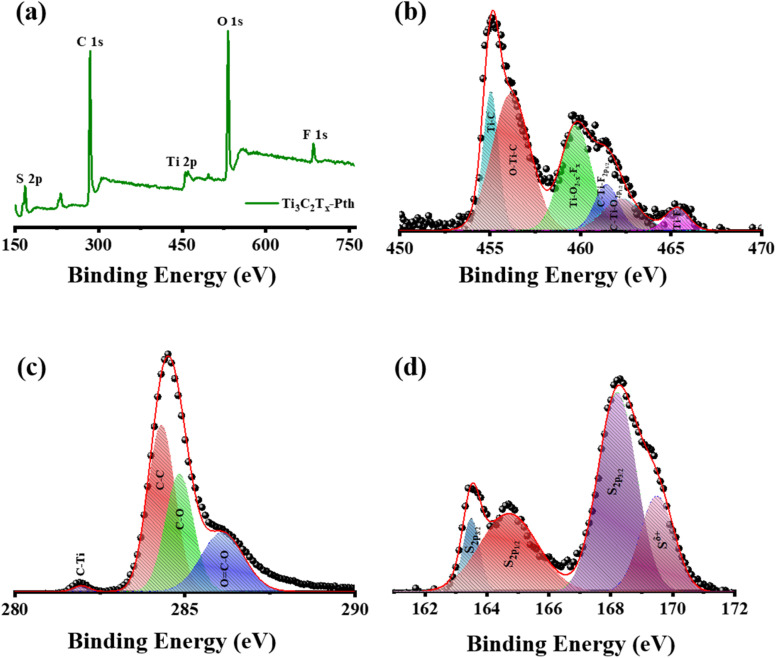
(a) XPS survey spectrum of Ti_3_C_2_T_*x*_-Pth, and XPS patterns of (b) Ti 2p, (c) C 1s, and (d) S 1s.

X-ray Photoelectron Spectroscopy (XPS) was employed to delve into the bonding states and surface chemical composition of the Ti_3_C_2_T_*x*_-PTh hybrid, revealing significant insights as observed in Fig. 4(a)–(d). The XPS spectra exhibited notable peaks for Ti 2p, C 1s, S 2p, O 1s, and F 1s, evidencing the successful amalgamation of Ti_3_C_2_T_*x*_ within the Ti_3_C_2_T_*x*_-PTh composite. These peaks were indicative of the –O, –OH, and –F functional groups, marking the composite's successful chemical integration. Furthermore, the presence of C 1s and S 2p peaks affirmed the synthesis of the Ti_3_C_2_T_*x*_-PTh CE. In the S 2p XPS spectrum, binding energies at 163.9 eV and 168.2 eV were observed for the S 2p_3/2_ state, and at 164.7 eV for the S 2p_1/2_ state, alongside a peak at 169 eV indicating S in a positively charged state (S^δ+^).^41,42^ This delineates the integration of thiophene units within the polymer chains, suggestive of polariton and dipolariton formation.^43^ The Ti 2p XPS spectrum unveiled six distinct peaks between 455 eV, 456.2 eV, 459.7 eV, 461.4 eV, 462.3 eV, and 465.3 eV, each reflecting different titanium bonding within the composite, indicative of Ti–C, C–Ti–O, Ti–O_2−*x*_ F_*x*_, C–Ti–F_2p_1/2__, C–Ti–O_2p_1/2__ and Ti–F_*x*_.^44,45^ The C 1s spectrum highlighted four peaks, signifying various carbon bonding such as C–Ti, C–C, and C–O, and O=C–O further elucidating the composite's chemical complexity at 281.6 eV, 284.8 eV, 286.2 eV, and 288 eV.^44^ The O 1s XPS spectrum, with peaks at 531.5 eV and 532.8 eV, pointed to diverse oxygen-containing functional groups such as C–Ti–(OH)_*x*_ and C=O, while the F 1s spectrum, with peaks at 685 eV and 685.7 eV, confirmed the presence of Ti–F and C–F bonds as observed in Fig. S3(a) and (b).†^46^ These results not only verify the PTh integration within the Ti_3_C_2_T_*x*_-PTh composite but also shed light on the composite's complex chemical and bonding nature, establishing a foundational understanding for further exploration of its attributes and potential applications.

In the quest to delineate the catalytic capabilities and durability of CEs for DSSCs, a methodical investigation was conducted using cyclic voltammetry (CV) within a three-electrode system. This experimental array comprised a platinum (Pt) wire and an Ag/AgCl electrode, serving as the counter and reference electrodes respectively, immersed in an electrolytic medium of 0.01 M LiI, 1.0 mM I_2_, and 0.1 M LiClO_4_. The procedure entailed performing CV sweeps in a defined potential from −0.7 to +1.3 V aimed at uncovering the redox dynamics exhibited by the electrodes. The primary focus was to scrutinize the electrochemical attributes of novel counter electrodes in comparison with conventional Pt-CE, with particular emphasis on the redox transformations involving triiodide to iodide and *vice versa* at a scanning rate of 50 mV s^−1^. The CV analysis of the Ti_3_C_2_T_*x*_-Pth revealed two distinct sets of redox peaks, where the first set was indicative of the I_3_^−^/I^−^ redox process and the latter represented the I_2_/I_3_^−^ process as given in eqn (1) and (2). The anodic peak observed at higher potentials corresponds to the oxidation of I_2_ to I^−^_3_ as described in eqn (2). Conversely, the cathodic peak at lower potentials corresponds to the reduction of I^−^_3_ to I^−^ as outlined in eqn (1). The cathodic peak associated with eqn (1) demonstrates the counter electrode's (CE) catalytic efficiency in reducing triiodide to iodide, represented by the cathodic peak current density. This observation accentuates the Ti_3_C_2_T_*x*_-Pth CE's adeptness in catalysing the reduction of I_3_^−^ to I^−^, a pivotal reaction within DSSCs.^47^

1 I_3_^−^ + 2e^−^ → 3I^−^

2 3I_2_ + 2e^−^ → 2I_3_^−^

Two distinctive characteristics, such as peak to peak separation (*E*_pp_) and peak current density, are used to determine counter electrode catalytic capacity. The peak-to-peak separation can be determined using eqn (3).

3 *E*_pp_ = |*E*_p_(anodic) − *E*_p_(cathodic)|

**Fig. 5:**
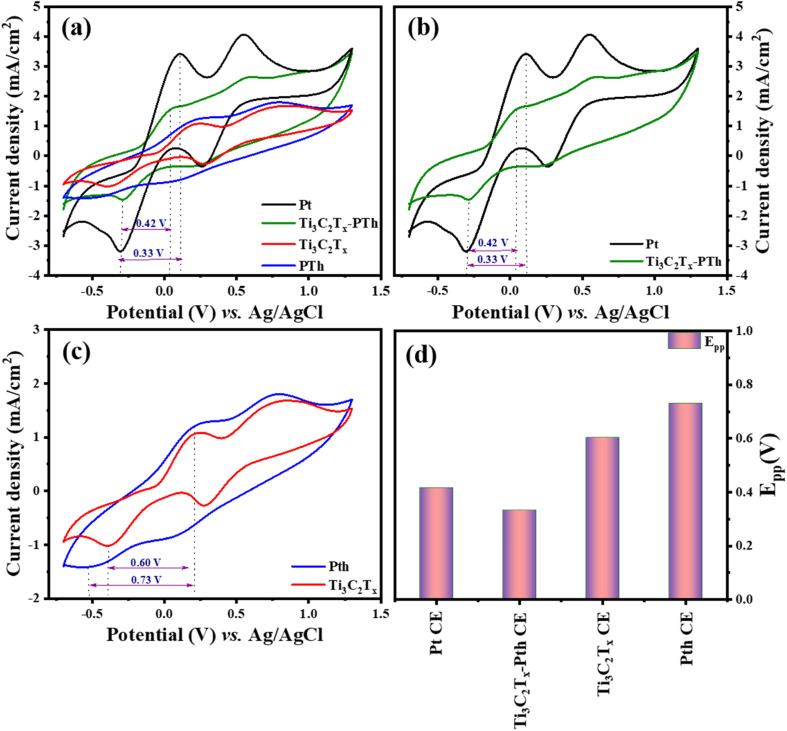
Cyclic voltammograms of (a) Pt, Ti_3_C_2_T_*x*_, Pth, Ti_3_C_2_T_*x*_-Pth at 50 mV s^−1^ scan rate (b–d) comparison of cyclic voltammograms Pt and Ti_3_C_2_T_*x*_-Pth CEs, Ti_3_C_2_T_*x*_ and Pth CEs, and overall *E*_pp_ comparison of Pt, Ti_3_C_2_T_*x*_, Pth, Ti_3_C_2_T_*x*_-Pth CEs.

In evaluating the electrochemical parameters of various CEs, the Ti_3_C_2_T_*x*_-Pth CE was identified as exhibiting superior peak current density and a diminished peak-to-peak separation potential (*E*_pp_) when compared with other alternative CEs. Remarkably, the Ti_3_C_2_T_*x*_-Pth CE demonstrated a peak current density of 1.62 mA cm^−2^ and an *E*_pp_ of 0.33 V, surpassing the electrochemical performance of both pristine Ti_3_C_2_T_*x*_ (1.08 mA cm^−2^, 0.60 V) and pristine Pth (1.24 mA cm^−2^, 0.73 V), while paralleling that of Pt CE (3.4 mA cm^−2^, 0.42 V) as observed in Fig. 5(a)–(d). These findings illuminate the reversible conversion of I_3_^−^ to I^−^, and superior electrocatalytic activity of the Ti_3_C_2_T_*x*_-PTh CE. The performance of Ti_3_C_2_T_*x*_ CE alone, albeit slightly lower in peak current density compared to PTh, but with a reduced *E*_pp_ suggests a synergistic amplification upon its integration with polythiophene. This synergy substantially uplifts its electrocatalytic potential towards iodide/tri-iodide electrolytes, leading to a significant improvement in PCE for DSSCs equipped with Ti_3_C_2_T_*x*_-PTh CEs.

**Fig. 6:**
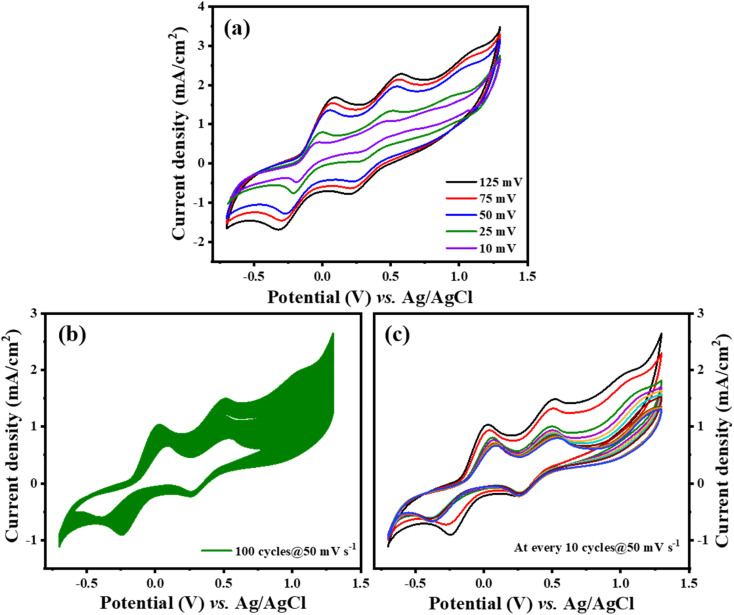
(a) Ti_3_C_2_T_*x*_-PTh at various scan rates (b) and (c) stability test of Ti_3_C_2_T_*x*_-PTh at 50 mV s^−1^ for 100 cycles and plotted at every 10 cycles.

As demonstrated in Fig. 6(a), there is a notable increase in peak current density concomitant with rising scan rates, signifying an enhancement in the catalytic efficiency of Ti_3_C_2_T_*x*_-PTh CE as the scan rate increases. The observation of a uniform, distortion-free pattern throughout these measurements implies a commendable level of reaction reversibility. Furthermore, as seen in Fig. 6(b), Ti_3_C_2_T_*x*_-PTh CE's electrochemical endurance was evaluated by subjecting it to CV for 100 cycles at a scan rate of 50 mV s^−1^ which was replotted at every 10 cycles as given in Fig. 6(c). The remarkable electrochemical stability of the CE was highlighted by the findings, which showed a steady cathodic peak current with no discernible changes in the CV profiles over the test period, suggesting its viability for prolonged use in DSSCs.

**Fig. 7:**
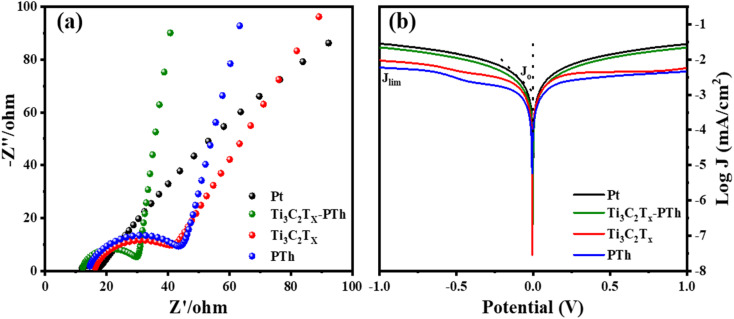
(a) Electrochemical impedance spectra of Pt, Ti_3_C_2_T_*x*_, PTh and Ti_3_C_2_T_*x*_-PTh CEs made from the symmetrical cell (b) Tafel polarisation of Pt, Ti_3_C_2_T_*x*_, PTh and Ti_3_C_2_T_*x*_-PTh CEs.

Electrochemical impedance spectroscopy (EIS) was utilized to understand the electrocatalytic behavior of the CEs further. The Nyquist plots of the CEs across a frequency range from 0.1 Hz to 100 000 Hz are depicted in Fig. 7(a), with the relevant parameters outlined in Table 1. The high-frequency region of the Nyquist plots showcases a semicircle that corresponds to the charge transfer resistance (*R*_ct_) at the counter electrode's interface, which is crucial in assessing the counter electrode's catalytic efficiency in reducing triiodide ions.^47^ The inclusion of Ti_3_C_2_T_*x*_ into the PTh CE was observed to decrease the *R*_ct_ value from 29.1 Ω to 17.6 Ω, under identical conditions. Additionally, the internal series resistance (*R*_s_), which stems from the combined resistance of the electrolyte and the electrode's sheet resistance, is discernible from the high-frequency real axis intercept in the EIS plots. The *R*_s_ for the Ti_3_C_2_T_*x*_-PTh CE was recorded at 12.2 Ω, showing a reduction from the 14.66 Ω observed for the solely PTh-based CE and 16.38 Ω for Ti_3_C_2_T_*x*_ CE. This decline in *R*_ct_ and *R*_s_ suggests an enhanced electrocatalytic performance of the composite electrode over the PTh CE, attributed to the composite electrode's larger electrochemical active surface area enhancing the charge transfer at the Ti_3_C_2_T_*x*_-PTh counter electrode/electrolyte interface, thereby accelerating the reduction reaction.^48^ In comparison, the Pt CE exhibited significantly lower *R*_ct_ (5.2 Ω) and *R*_s_ (11.9 Ω) values than those of the Ti_3_C_2_T_*x*_-PTh CE. Given the influence of *R*_s_ and *R*_ct_ on the fill factor (FF) of DSSCs, Pt was shown to achieve a superior FF, as demonstrated in the photovoltaic efficiency tests outlined in Table 1.

**Table 1:** PV and EIS performance of the DSSCs constructed using Pt CE, Ti_3_C_2_T_*x*_ CE, PTh CE, and Ti_3_C_2_T_*x*_-PTh CE tested under 100 mW cm^−2^

CEs	*R*_s_ (Ω)	*R*_ct_ (Ω)	*V*_oc_ (V)	*J*_sc_ (mA cm^−2^)	Fill factor	*η*
Pt	12	5.2	0.66	19.27	62.7	7.9
Ti_3_C_2_T_*x*_	16.38	25.2	0.66	12.6	55.3	4.61
Pth	14.6	29.1	0.66	11.6	55.5	4.19
Ti_3_C_2_T_*x*_-Pth	12.2	17.6	0.67	15.54	56.05	5.82

**Fig. 8:**
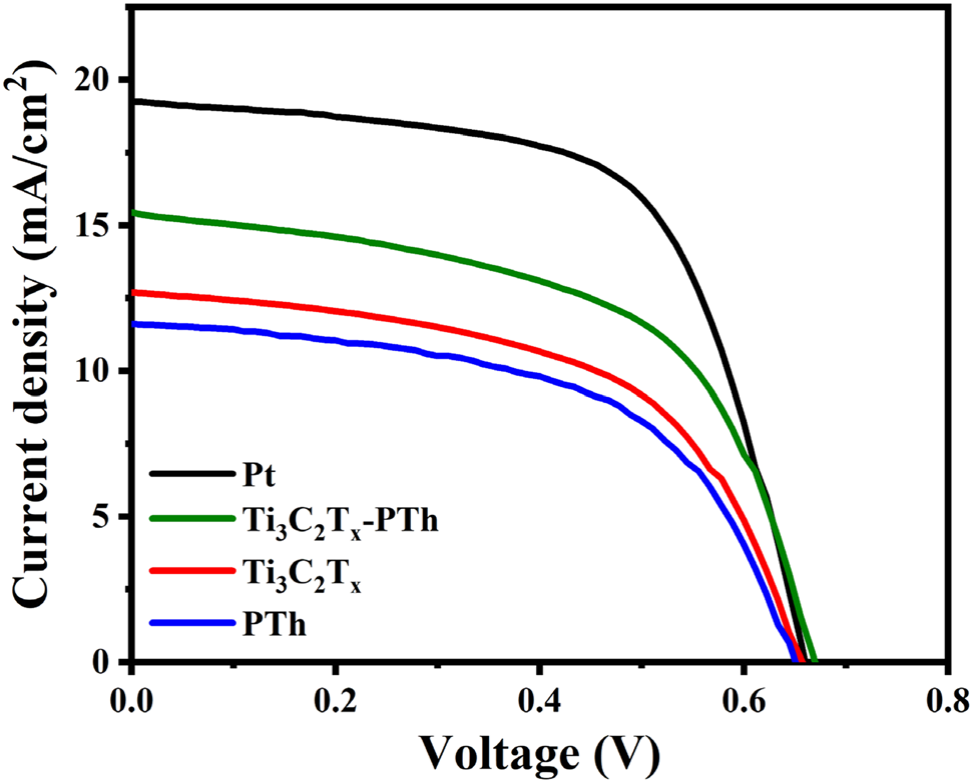
*J*–*V* photocurrent density–voltage curves of DSSC fabricated with Pt, Ti_3_C_2_T_*x*_, PTh, and Ti_3_C_2_T_*x*_-PTh CEs.

The Tafel polarization analysis is utilized as an efficacious method for investigating electrochemical behaviors. Fig. 7(b) delineates the Tafel testing results for varied counter electrodes (CEs), highlighting two pivotal parameters: the exchange current density (*J*_o_) and the maximal diffusion current density (*J*_lim_). Elevated values for these parameters are indicative of superior catalytic performance and enhanced electron diffusion rate.^49,50^ As represented in Fig. 7(b), the CE with Ti_3_C_2_T_*x*_-PTh composition exhibits the most prominent *J*_o_ and *J*_lim_ values in the Tafel and diffusion segments, measured at −2.8 mA cm^−2^ and −1.6 mA cm^−2^, respectively. These values are higher than those of pristine Ti_3_C_2_T_*x*_ CE, which have *J*_o_ and *J*_lim_ values of −2.9 mA cm^−2^ and −2.0 mA cm^−2^, and PTh CE, which have values of −3.0 mA cm^−2^ and −2.2 mA cm^−2^. Additionally, these values are comparable to those of Pt CE, which has *J*_o_ and *J*_lim_ values of −2.5 mA cm^−2^ and −1.5 mA cm^−2^. The observed increase in catalytic efficiency can be attributed to the combined effect of the abundant active sites afforded by Ti_3_C_2_T_*x*_ and PTh and the efficient electron transport channels. Moreover, the relationship for *J*_o_ as specified in eqn (4) is identified to be inversely proportional to the charge transfer resistance (*R*_ct_), thereby aligning with the observations derived from the Electrochemical Impedance Spectroscopy (EIS) studies.4
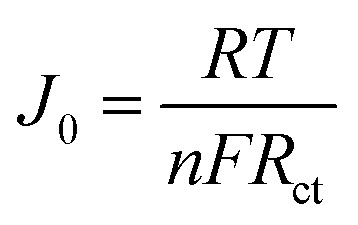
In Fig. 8, the *J*–*V* characteristic profiles of DSSCs utilizing different CEs are illustrated, with Table 1 providing a summary of the pertinent metrics. The analysis of *J*–*V* curves reveals that DSSCs equipped with a Ti_3_C_2_T_*x*_-PTh CE achieved a PCE (*η*) of 5.8%. This efficiency closely rivals that of cells with a Pt CE, recorded at 7.9%, and markedly outperforms the efficiencies of cells with individual CEs. The Ti_3_C_2_T_*x*_-PTh CE demonstrates superior performance over pure PTh CE and pure Ti_3_C_2_T_*x*_ CE in terms of short-circuit current density (*J*_sc_, 15.5 mA cm^−2^), open-circuit voltage (*V*_oc_, 0.67 V), and fill factor (FF, 56%). While the Pt electrode displays a higher *V*_oc_ (0.66 V) and FF (63%), its *J*_sc_ (19.3 mA cm^−2^) is marginally superior to that of the composite electrode. The enhancement in performance metrics with the Ti_3_C_2_T_*x*_-PTh CE is ascribed to its augmented surface area and improved electrocatalytic activity, owing to the integration of conductive Ti_3_C_2_T_*x*_ sheets. This configuration facilitates a more efficient reduction of I_3_^−^. The PCE of the Ti_3_C_2_T_*x*_-PTh CE exhibited superior performance compared to other reported 2D counter electrode materials as given in Table 2. The superior *V*_oc_ and FF observed with the Pt electrode stems from its exceptional electronic conductivity and diminished charge transfer resistance, which expedite the I_3_^−^ reduction process. The enhancement in photovoltaic performance of DSSCs *via* the utilization of Ti_3_C_2_T_*x*_-PTh CE is primarily ascribed to the synergistic catalytic effects emanating from the integration of Ti_3_C_2_T_*x*_ and PTh. This synergism significantly contributes to an elevation in *J*_sc_ to 15.54 mA cm^−2^ and FF to 56%.

**Table 2:** Comparison of photovoltaic performance of DSSC fabricated with Ti_3_C_2_T_*x*_-Pth CE and other reported works

Counter electrodes	*J*_sc_ (mA cm^−2^)	*V*_oc_ (V)	Fill factor	*η*	References
PTh-IrO_2_	10.1	0.6	50	3.01	51
Co-SnS_2_/rGo	11.85	0.77	60.1	5.54	52
MoSe_2_	16.28	0.72	41	4.8	53
Carbon black/SnSe	12.4	0.66	62	5.0	54
MXene-rGO	10.9	0.64	62	4.36	55
V_2_C	8.07	0.76	53	3.39	56
MXene-MoS_2_	11.4	0.64	71	5.21	55
Ti_3_C_2_T_*x*_-Pth	15.54	0.67	56.05	5.82	This work

To further elucidate the photovoltaic characteristics of DSSCs, the external quantum efficiency (EQE) spectra of DSSCs employing Pt and Ti_3_C_2_T_*x*_-PTh CEs are presented in Fig. S4.† The utilization of the same dye results in a consistent spectral response range across all DSSCs. The EQE for DSSCs with Pt CE and Ti_3_C_2_T_*x*_-PTh CE peaks at 540 nm, attaining values of 41% and 38%, respectively, which are in close proximity to the performance exhibited by Pt CE. This enhancement in photovoltaic performance is attributable to the synergistic interaction of multiple active sites, superior charge transfer dynamics, and enhanced electrocatalytic activity intrinsic to the Ti_3_C_2_T_*x*_-PTh CE.

## 4. Conclusions

The Ti_3_C_2_T_*x*_-PTh composite was synthesized through a liquid–liquid interfacial polymerization technique, subsequently serving as an alternative CE in DSSCs, aiming to replace the Pt CE. Analyses utilizing XRD, FTIR, and XPS confirmed the effective integration of Ti_3_C_2_T_*x*_ into the PTh matrix. The morphological analysis revealed a consistent dispersal of Ti_3_C_2_T_*x*_ within the polymer framework. Compared to the pristine Ti_3_C_2_T_*x*_ CE and PTh CE, the composite electrode exhibited superior electrocatalytic performance, especially in the reduction of triiodide/iodide (I_3_^−^/I^−^), primarily due to its increased active surface area and enhanced charge transport capabilities. The DSSCs with the Ti_3_C_2_T_*x*_-PTh CE demonstrated a higher short-circuit current density (*J*_sc_) and fill factor (FF) compared to devices using pure PTh. This improvement is attributed to the inclusion of Ti_3_C_2_T_*x*_ sheets, which notably enhances electron transport within the PTh matrix by decreasing resistance. Investigation of the photovoltaic performance of the DSSC constructed using Ti_3_C_2_T_*x*_-PTh CE revealed a PCE of 5.82%, comparable to devices equipped with Pt CE. Therefore, the Ti_3_C_2_T_*x*_-PTh CE material emerges as a viable and effective substitute for the costly Pt CE, marking its potential for future generation CE in DSSCs. This facile synthesis approach could significantly contribute to the development of other Ti_3_C_2_T_*x*_-based composite materials for use in economical and efficient photoelectronic devices in the foreseeable future.

## Data availability

The authors declare that the data supporting the findings are available within the article.

## Conflicts of interest

The authors declare no competing conflict of interest.

## Supplementary Material

RA-014-D4RA02651A-s001

## References

[cit1] Kokkonen M., Talebi P., Zhou J., Asgari S., Soomro S. A., Elsehrawy F., Halme J., Ahmad S., Hagfeldt A., Hashmi S. G. (2021). J. Mater. Chem. A.

[cit2] Hasan A. M. M., Susan M. A. B. H. (2024). RSC Adv..

[cit3] Noman M., Khan Z., Jan S. T. (2024). RSC Adv..

[cit4] O'regan B., Grätzel M. (1991). Nature.

[cit5] Gómez L. J. V., Iglesias A. L., Soto V. M., Sarabia A. O., Castro R. V., Maldonado E. A. L., Guzmán M. T. O., Soto C. A. R., Medina E. G. L., Arce J. L. V. (2023). RSC Adv..

[cit6] V Flint H., Tito H. A. R., James R. D., Cucinotta F., Gibson E., Caceda M. E. Q. (2024). RSC Adv..

[cit7] Aftabuzzaman M., Ahmed M. S., Matyjaszewski K., Kim H. K. (2022). Chem. Eng. J..

[cit8] Hu Z., Li Y., Li A., Wang H.-H., Wang X.-F. (2023). RSC Adv..

[cit9] Wu C., Li R., Wang Y., Lu S., Lin J., Liu Y., Zhang X. (2020). Chem. Commun..

[cit10] Chen H.-H., Lin P.-C., Tsai H.-E., Tsao W.-Y., Wang C.-L. (2022). J. Electroanal. Chem..

[cit11] Pang Z., Zhao Y., Duan Y., Duan J., Tang Q., Yu L. (2019). J. Energy Chem..

[cit12] Mirzaei M., Gholivand M. B. (2022). Sol. Energy.

[cit13] Wu K., Liu S., Wu Y., Ruan B., Guo J., Wu M. (2022). Sol. Energy Mater. Sol. Cells.

[cit14] Gao C., Han Q., Wu M. (2018). J. Energy Chem..

[cit15] Kouhnavard M., Yifan D., D'Arcy J. M., Mishra R., Biswas P. (2020). Sol. Energy.

[cit16] Yao X., He B., Cui L., Ti J., Chen H., Duan Y., Tang Q. (2022). Catal. Commun..

[cit17] Kim S. J., Koh H.-J., Ren C. E., Kwon O., Maleski K., Cho S.-Y., Anasori B., Kim C.-K., Choi Y.-K., Kim J. (2018). ACS Nano.

[cit18] Naguib M. , Kurtoglu M., Presser V., Lu J., Niu J., Heon M., Hultman L., Gogotsi Y. and Barsoum M. W., in MXenes, Jenny Stanford Publishing, 2011, pp. 15–29

[cit19] Xu J., Shim J., Park J., Lee S. (2016). Adv. Funct. Mater..

[cit20] Lian P., Dong Y., Wu Z.-S., Zheng S., Wang X., Wang S., Sun C., Qin J., Shi X., Bao X. (2017). Nano Energy.

[cit21] Rajendran J. (2023). J. Hazard. Mater..

[cit22] Chakoma S., Pei X., Qin H., Ghandehari A., NajafiKhoshnoo S., Rajendran J., Esfandyarpour R. (2024). Nano Res..

[cit23] Lee S. W., Pei X., Rajendran J., Esfandyarpour R. (2022). IEEE J. Flex. Electron..

[cit24] Rajendran J., Sundramoorthy A. K., Ganapathy D., Atchudan R., Habila M. A., Nallaswamy D. (2022). J. Hazard. Mater..

[cit25] Li Z., Wang P., Ma C., Igbari F., Kang Y., Wang K.-L., Song W., Dong C., Li Y., Yao J. (2021). J. Am. Chem. Soc..

[cit26] Kaur N., Singh D. P., Mahajan A. (2022). J. Electron. Mater..

[cit27] Li Z., Li H., Wang S., Yang F., Zhou W. (2022). Chem. Eng. J..

[cit28] Chen X., Zhuang Y., Shen Q., Cao X., Yang W., Yang P. (2021). Sol. Energy.

[cit29] He Y., Shen Z., Yue G., Gao Y., Huo J., Dong C., Mao Y., Tan F. (2022). J. Alloys Compd..

[cit30] Nie F., Zhao H., Liu S., Li Y., Zhang H., Wu M., Wu K. (2024). Diamond Relat. Mater..

[cit31] Gund G. S., Park J. H., Harpalsinh R., Kota M., Shin J. H., Kim T., Gogotsi Y., Park H. S. (2019). Joule.

[cit32] Bora C., Sarkar C., Mohan K. J., Dolui S. (2015). Electrochim. Acta.

[cit33] Yang L., Dall'Agnese Y., Hantanasirisakul K., Shuck C. E., Maleski K., Alhabeb M., Chen G., Gao Y., Sanehira Y., Jena A. K. (2019). J. Mater. Chem. A.

[cit34] Shabeeba P., Thayyil M. S., Pillai M. P., Soufeena P. P., V Niveditha C. (2018). Russ. J. Electrochem..

[cit35] Jiang Q., Lei Y., Liang H., Xi K., Xia C., Alshareef H. N. (2020). Energy Storage Mater..

[cit36] Luo W., Wei Y., Zhuang Z., Lin Z., Li X., Hou C., Li T., Ma Y. (2022). Electrochim. Acta.

[cit37] Barakzehi M., Montazer M., Sharif F., Norby T., Chatzitakis A. (2019). Electrochim. Acta.

[cit38] Bora C., Pegu R., Saikia B. J., Dolui S. K. (2014). Polym. Int..

[cit39] Boota M., Gogotsi Y. (2019). Adv. Energy Mater..

[cit40] Wang Y., Ding Y., Guo X., Yu G. (2019). Nano Res..

[cit41] Liang H., Li X. (2009). Appl. Catal., B.

[cit42] Dong C., Li D., Wang H., Cai B., Xin Y., Peng H., Zhao Y., Wang N., Cui Z., Wang G. (2023). Carbon.

[cit43] Xue J., Shi Y., Wang W., Yu Y., Tang C. (2022). J. Mater. Sci.: Mater. Electron..

[cit44] Ghidiu M., Halim J., Kota S., Bish D., Gogotsi Y., Barsoum M. W. (2016). Chem. Mater..

[cit45] Rakhi R. B., Ahmed B., Hedhili M. N., Anjum D. H., Alshareef H. N. (2015). Chem. Mater..

[cit46] Kang R., Zhang Z., Guo L., Cui J., Chen Y., Hou X., Wang B., Lin C.-T., Jiang N., Yu J. (2019). Sci. Rep..

[cit47] Gong F., Xu X., Zhou G., Wang Z.-S. (2013). Phys. Chem. Chem. Phys..

[cit48] Liu C.-Y., Huang K.-C., Chung P.-H., Wang C.-C., Chen C.-Y., Vittal R., Wu C.-G., Chiu W.-Y., Ho K.-C. (2012). J. Power Sources.

[cit49] Jeong H., Kim J.-Y., Koo B., Son H. J., Kim D., Ko M. J. (2016). J. Power Sources.

[cit50] Gopi C. V. V. M., Venkata-Haritha M., Ravi S., Thulasi-Varma C. V., Kim S.-K., Kim H.-J. (2015). J. Mater. Chem. C.

[cit51] Asok A., Haribabu K. (2023). Curr. Appl. Phys..

[cit52] Raveena J., Chandrapal R. R., Bakiyaraj G., Manikandan V. S., Athitya S., Archana J., Navaneethan M. (2023). Mater. Today Commun..

[cit53] Ahmad K., Kim H. (2023). Mater. Chem. Phys..

[cit54] Zatirostami A. (2021). Thin Solid Films.

[cit55] Gasso S., Mahajan A. (2022). Chem. Phys. Lett..

[cit56] Xu C., Zhao X., Sun M., Ma J., Wu M. (2021). Electrochim. Acta.

