# Supplementary material for: Fabrication of a free-standing Ti3C2Tx-PTh counter electrode via interfacial polymerization for dye-sensitized solar cells
Source: RSC Adv. 2024 Jul 31;14(33):24000–9. doi: 10.1039/d4ra02651a (PMC11289875; doi:10.1039/d4ra02651a)
Supplement: RA-014-D4RA02651A-s001 [file RA-014-D4RA02651A-s001.pdf]

Supplementary Information (SI) for RSC Advances.
This journal is © The Royal Society of Chemistry 2024

SUPPLEMENTARY INFORMATIONS

Fabrication of a free-standing $Ti_3C_2T_x$-PTh counter electrode via interfacial polymerization for dye-sensitized solar cells

Suruthi Priya Nagalingam[a], Saravanan Pandiaraj[b], Khalid E. Alzahrani[c], Abdullah. N Alodhayb[c] and Andrews Nirmala Grace[a*]

[a] Centre for Nanotechnology Research, Vellore Institute of Technology, Vellore- 632014

[b]Department of Self-Development Skills, King Saud University, Riyadh, 11451, Saudi Arabia.

[c]Department of Physics and Astronomy, College of Science, King Saud University, Riyadh, 11451, Saudi Arabia.

*Corresponding Author mail: anirmalagladys@gmail.com

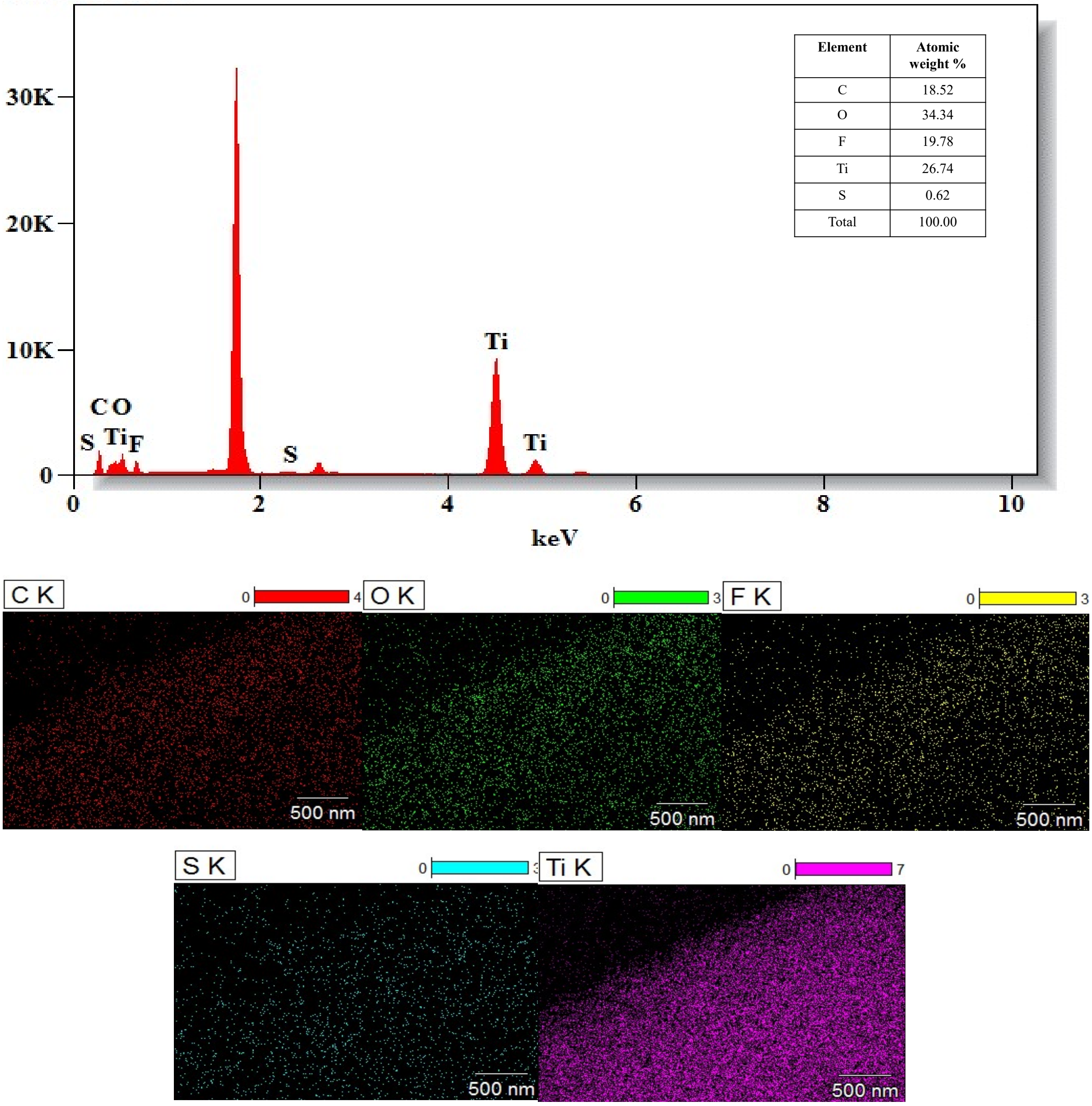

Fig S1. EDS spectrum and elemental mapping of $Ti_3C_2T_x$-PTh

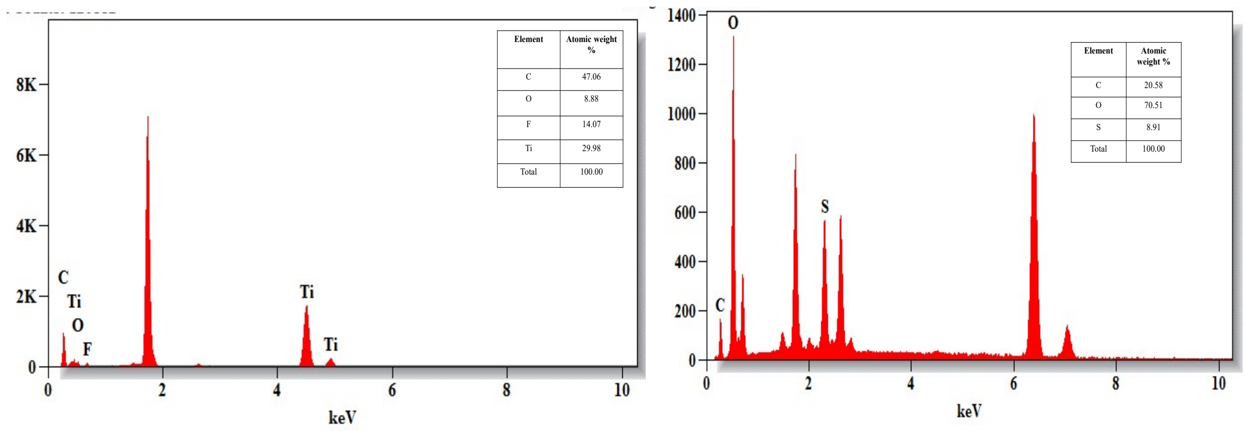

Fig S2. EDS spectrum of pristine PTh and $Ti_3C_2T_x$

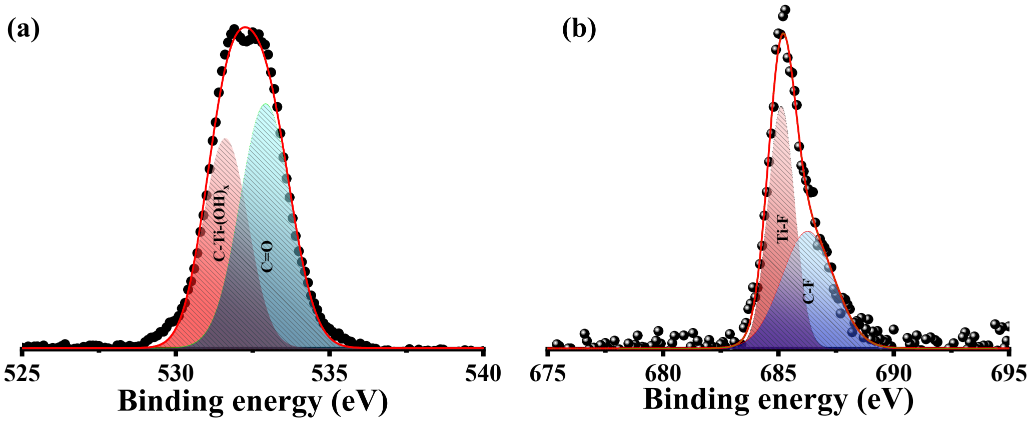

Fig S3. XPS patterns of (a) O 1s and (b) F 1s

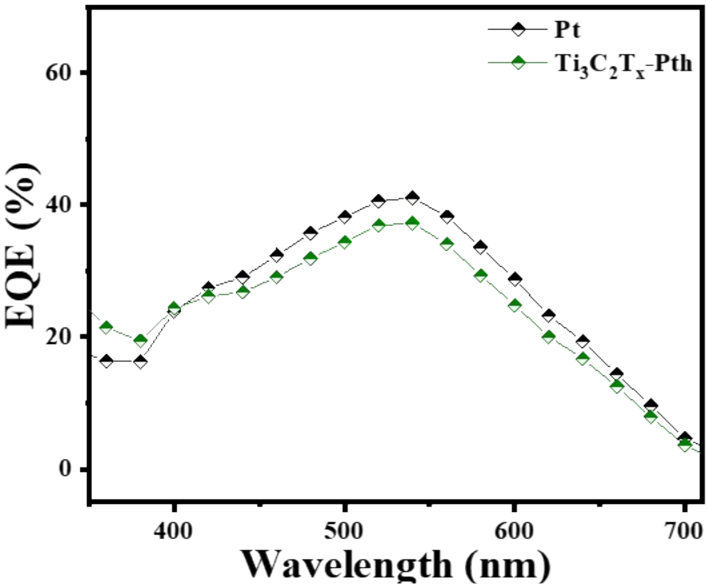

Fig S4. EQE of DSSCs with Pt and $Ti_3C_2T_x$-Pth CEs
